# Ceftazidime/avibactam Improves the Antibacterial Efficacy of Polymyxin B Against Polymyxin B Heteroresistant KPC-2-Producing *Klebsiella pneumoniae* and Hinders Emergence of Resistant Subpopulation *in vitro*

**DOI:** 10.3389/fmicb.2019.02029

**Published:** 2019-09-03

**Authors:** Xingyan Ma, Yuting He, Xuegao Yu, Yimei Cai, Jianming Zeng, Renxin Cai, Yang Lu, Liang Chen, Cha Chen, Bin Huang

**Affiliations:** ^1^Department of Laboratory Medicine, The First Affiliated Hospital, Sun Yat-sen University, Guangzhou, China; ^2^Department of Laboratory Medicine, The Second Affiliated Hospital, Guangzhou University of Chinese Medicine, Guangzhou, China; ^3^Department of Laboratory Medicine, Guangdong Provincial Hospital of Chinese Medicine, Guangzhou, China; ^4^Public Health Research Institute Tuberculosis Center, New Jersey Medical School, Rutgers University, Newark, NJ, United States

**Keywords:** ceftazidime/avibactam, polymyxin B, heteroresistance, KPC-2-producing *Klebsiella pneumoniae*, bacterial killing activity

## Abstract

Due to the increasing multidrug resistance and limited antibiotics, polymyxin B revived as the last resort for the treatment of carbapenemase-producing *Klebsiella pneumoniae* (CRKP). Unfortunately, the heteroresistance hampers polymyxin B monotherapy treatment via the amplification of resistant subpopulation. Reliable polymyxin B based combinations are demanded. Ceftazidime/avibactam has been regarded as a new salvage therapy against CRKP. The occurrence of heteroresistance was confirmed by population analysis profiling (PAP). Our study demonstrated that polymyxin B and ceftazidime/avibactam combinations improved the *in vitro* antimicrobial activity of polymyxin B and delayed or suppressed the regrowth of resistant subpopulation by time-kill studies. Ceftazidime/avibactam at around MIC values (0.5–1 × MIC) plus clinically achievable concentrations of polymyxin B (0.5–2 mg/L) resulted in sustained killing against polymyxin B-heteroresistant isolates. Active PmrAB and PhoPQ systems and a *pmrA* mutation (G53R) in resistant subpopulation might associate with heteroresistance, but further investigation was required. Our findings suggested that the heteroresistance represented barriers to polymyxin B efficacy, and the combination of polymyxin B with ceftazidime/avibactam could be potentially valuable for the treatment of heteroresistant CRKP. Further, *in vivo* studies need to be performed to evaluate the efficacy of this combination against heteroresistant strains.

## Introduction

The global spread of carbapenemase-producing *Klebsiella pneumoniae* (CRKP) posed a severe challenge to public health, especially KPC-producing *K. pneumoniae* (KPC-Kp) (Willyard, 2017; Decraene et al., 2018; Gu et al., 2018). To date, available options for CRKP were limited. Therefore, polymyxin B (PMB) revived as one of the last-resort options for CRKP (Falagas and Michalopoulos, 2006; Landman et al., 2008). However, there are some challenges when clinicians use polymyxin B, such as its toxicities, unreliable plasma concentrations, and several issues with polymyxin B susceptibility testing (Ezadi et al., 2019). Additionally, bacteria employed several strategies to survive to polymyxins, including LPS modifications by activation of the two-component systems (TCSs), particularly modifications of lipid A, the efflux pumps, and plasmid-mediated resistance (Ma et al., 2018; Meletis and Skoura, 2018).

Significantly, the heteroresistance raised a diagnostic and therapeutic dilemma for clinicians, which the resistant subpopulations in heteroresistant strains were undetectable and could affect the clinical outcome (El-Halfawy and Valvano, 2015; Band and Weiss, 2019). El-Halfawy and Valvano (2015) recommended defining heteroresistance as subpopulations of an isogenic strain exhibit widely various susceptibilities to a particular antimicrobial agent, i.e., when the lowest concentration exhibiting maximum inhibition is eightfold higher than the highest non-inhibitory concentration in terms of population analysis profiling (PAP). However, this method is too laborious and complex to apply to clinical detection. Increasing studies have demonstrated that conventional susceptibility tests could misclassify heteroresistant strains as susceptible and might lead to clinical treatment failure (Band et al., 2018; Turlej-Rogacka et al., 2018; Ezadi et al., 2019). But little work has been done to evaluate the efficacy of available antibiotics against heteroresistant strains. Ceftazidime/avibactam, a β-lactam/β-lactamase inhibitor combination, has been proposed as a new salvage therapy for severe KPC-Kp infections (Barber et al., 2018; Manning et al., 2018; Tumbarello et al., 2019). The objective of this study was to evaluate the *in vitro* effect of ceftazidime/avibactam in combination with polymyxin B against polymyxin B heteroresistance *Klebsiella pneumoniae*.

## Materials and Methods

### Bacterial Strains and Characterization

Seventeen non-duplicate clinical isolates were obtained from two tertiary hospitals in Guangzhou from 2013 to 2014, as shown in Supplementary Table S1. All isolates were stored at −80°C and subcultured onto blood agar plate before each experiment. All isolates were reconfirmed by matrix-assisted laser desorption ionization–time of flight mass spectrometry (MALDI-TOF). Carbapenemase genes, ESBLs genes, outer member protein genes, and *mcr-1* gene were amplified by primers described previously and then sequenced by Sanger sequencing (Pagani et al., 2003; Poirel et al., 2011; Liu et al., 2016).

### Antimicrobials and Antimicrobial Susceptibility Testing

Polymyxin B (Sigma-Aldrich, United States), ceftazidime hydrate (Sigma-Aldrich, United States) and avibactam (MedChem Express, United States) were freshly prepared for each experiment and filter sterilized using a 0.22 μm filter. Avibactam was tested at a fixed concentration of 4 mg/L (Clinical and Laboratory Standards Institute [CLSI], 2017). Mueller-Hinton broth (Oxoid, United Kingdom) supplemented with calcium and magnesium (25.0 mg/liter Ca^2+^ and 12.5 mg/liter Mg^2+^) (CAMHB) and Mueller-Hinton II agar (Oxoid, United Kingdom) were used for susceptibility testing and all *in vitro* models. The breakpoints for polymyxin B, ceftazidime and ceftazidime/avibactam were defined by CLSI-M100-S26. Quality control was monitored with *Escherichia coli* strains ATCC 25922 and *Klebsiella pneumoniae* strain ATCC 700603.

### Polymyxins Population Analysis Profiles (PAPs)

Population analysis profilings were performed to investigate the presence of polymyxin B heteroresistance in duplicate (Nicoloff et al., 2019). Fifty-microliter of dilutions of an overnight culture (∼10^8^ CFU/ml) were plated on Mueller-Hinton agar plates containing polymyxin B at the following concentrations: 0.5, 1, 2, 4, 8, 16, and 32 mg/L. After overnight incubation at 37°C, colonies were counted. Agar plate preparation followed CLSI M7-09 documents for MIC determination by agar dilution assays. The detection limit of PMB resistant subpopulations was 20 CFU/ml.

### To Measure the Stability of Resistant Subpopulation

After overnight growth of B1, D1, and D4 (without polymyxin B), fifty-microliter aliquots of the overnight culture were added into tubes with 16 mg/L polymyxin B. After serially diluted, suspension was plated on M-H agar plates with and without 16 μg/ml polymyxin B to count CFU of total, and resistant subpopulation at desired time points (day 1). A subculture (1:100) was grown overnight in CAMHB without 16 μg/ml polymyxin B, serially diluted, and plated on M-H agar with or without 16 μg/ml polymyxin B to count CFU of parental, and resistant cells (day 2). Repeated this process in CAMHB broth without antibiotics (day 3 and 4).

### Genes Expression Analysis

The polymyxin B-resistant subpopulations were collected from the last step. Cultures of parental strains and resistant subpopulations were grown in CAMHB medium without polymyxin B at 37°C with shaking to an OD600 of 0.5. The mRNA of strains was extracted by Trizol method. By the process of RT-PCR using the PrimeScript^TM^ RT reagent Kit with gDNA Eraser (TAKARA, China), the cDNAs were got. Then the *phoP*, *phoQ*, *mgrB*, *pmrA*, *pmrB*, *pmrC*, and *acrB* gene expression were detected through quantitative real-time PCR (qRT-PCR) using the SYBR^®^ Premix Ex Taq^TM^ II (Tli RNaseH Plus) kit (TAKARA, China), as previously described (Jayol et al., 2015). Each experiment was performed in triplicate. The expression of target genes was normalized relative to the RNA polymerase beta subunit gene *rpoB*. Threshold cycle (Ct) numbers were confirmed by the qRT-PCR system software, and data was analyzed in accordance with the 2^–ΔΔCt^ method. The expression levels of the target genes were compared with those of *K. pneumoniae* ATCC 700603 (polymyxin B susceptible strain, expression = 1).

### Whole-Genome Sequencing and SNPs Analysis

The whole genome sequencing of twenty-four carbapenem-resistant isolates was performed with a NextSeq 500 platform (Illumina Inc., San Diego, CA, United States). Briefly, the genomic DNA was extracted using a MiniBEST Bacteria Genomic DNA Extraction Kit (Takara, Dalian, China). To prepare the DNA library for sequencing, a QIAseq FX DNA Library Kits (Qiagen Inc., Valencia, CA, United States) was used following the manufacturer’s recommendations. The quality and quantity of the libraries were assessed with LabChip GX (Perkin Elmer; Waltham, MA, United States) and Qubit dsDNA HS Assay Kit (Life Technologies, United States). All barcoded libraries were pooled together in equimolar amounts and each pool was sequenced on NextSeq 500 in PE-150 bp mode. Later, sequencing raw reads were processed for library adapter removal and filtering using FASTQ preprocessor Fastp v0.12.5 (Chen et al., 2018) and *de novo* assembly with SPAdes v3.13.0 (Bankevich et al., 2012). Genomic repeats were removed from the analyses by filtering out reads that mapped to multiple positions in *K. pneumoniae subsp. pneumoniae* HS11286 (NCBI accession number: NC_016845). Single nucleotide polymorphisms (SNPs) and insertions and deletions (indels) generated by Snippy.

### Synergy Testing Using the Checkerboard Assay and Time-Kill Assay

Time-kill studies were performed using a 5-ml time kill assay with an initial inoculum of ∼10^6^ CFU/ml suspended in CAMHB. Each experiment was performed in duplicate. Ceftazidime-avibactam concentrations of 0.25×, 0.5×, 1×, 2×, 4×, and 8× MIC and polymyxin B concentrations of 0.5, 1, 2, and 6 mg/L were evaluated as monotherapy. Meanwhile, avibactam was added to a final concentration of 4 mg/L. A 3-by-3 concentration matrix of ceftazidime-avibactam 0.25×, 0.5×, and 1× MIC) in combination with PMB (0.5, 1, and 2 mg/L) was evaluated. All concentrations evaluated were clinically achievable, and supratherapeutic concentrations were also selected to evaluate potential advantages of intensive dosing (Avedissian et al., 2019; Tumbarello et al., 2019). Samples were incubated with shaking (37°C, 200 rpm), and were obtained at 0, 4, 8, 12, and 24 h for quantification of bacteria. The change in log10 CFU per milliliter at time t (CFU_t_) compared to the baseline value (0 h) (CFU_0_) was the index of pharmacodynamic effect. A > 3 log10 CFU/ml reduction from baseline was considered as the bactericidal activity. Synergy was considered as a >2 log10 CFU/ml reduction and additivity as a >1 to <2 log10 reductions in CFU/ml caused by the combination of PMB and ceftazidime-avibactam compared to the most effective single antibiotic in the combination. Subsequently, the effects of combinations against heteroresistant strains were evaluated using microbroth checkerboard method. Given that we could not get clear MIC results of polymyxin B due to the presence of skip-wells, the concentrations of polymyxin B in the combinations were selected as these used in time-kill assay. And ceftazidime-avibactam in the combinations was two dilutions above and four dilutions below the MIC. The fractional inhibitory concentration index (FICI) was calculated using the following equation: FICI = FICA + FICB, where FICA = MIC of drug A in a combination/MIC of drug A alone, and FICB = MIC of drug B in a combination/MIC of drug B alone. The FICI results were interpreted as synergistic (≤0.5), additive (>0.5 to ≤1), or indifferent (>1).

### Statistical Analysis

Statistical analyses were performed using Prism 7 (GraphPad Software). The median was used to describe the average fold increase in heteroresistant strains. The two-tailed student’s *t*-test was used to analyze the significance of relative gene expression level between parental strain and resistant subpopulation.

### Accession Number

Sequence data from this study were deposited in NCBI’s short read archive (SRA) under project accession number PRJNA504930.

## Results

### Antimicrobial Susceptibility Testing and Heteroresistance Identification

**FIGURE 1:**
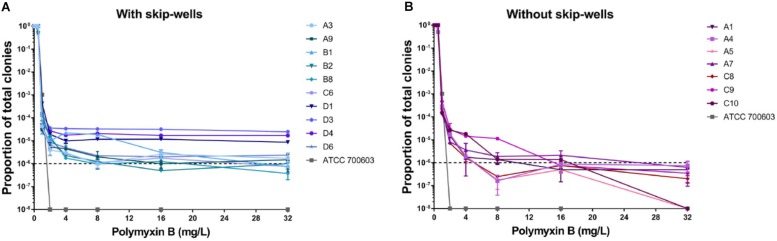
Population analysis profiles (PAPs) of all isolates were performed in duplicate. **(A)** The PAP curves of isolates with skip-wells. **(B)** The PAP curves of isolates without skip-wells.

In our study, all isolates belonged to ST 11 and harbored KPC-2, TEM, SHV, and CTX-M (Supplementary Table S1). All isolates remained sensitive to ceftazidime/avibactam (Supplementary Table S1). Seven isolates showed sensitive to polymyxin B with clear wells (range 0.06125–0.125 mg/L), but the presence skip-wells was observed in the other ten isolates (Supplementary Table S1). The results of PAPs indicated all isolates exhibited heteroresistant to polymyxin B. Most of our isolates (except C10 and A5) harbored minor resistant subpopulations able to withstand at least 32 mg/L polymyxin B (Figure 1). In contrast, susceptible strain (ATCC 700603) was entirely killed by 2 mg/L polymyxin B. Compared with the isolates without skip-wells, the frequencies of resistant subpopulation among isolates with skip-wells were higher (above 10^–6^) and remained constant with polymyxin B concentrations increasing.

**FIGURE 2:**
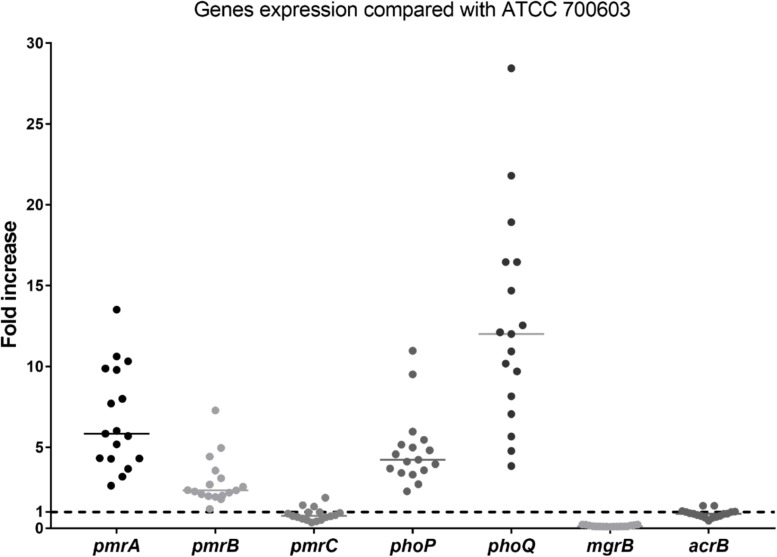
Relative gene expression, expressed as fold change, of the *phoP*, *phoQ*, *mgrB*, *pmrA*, *pmrB*, *pmrC*, and *acrB* genes in 17 clinical *Klebsiella pneumoniae* isolates. Expression levels were detected by qRT-PCR, with polymyxin B-susceptible *K. pneumoniae* ATCC 700603 used as the reference strain (expression = 1), which is indicated by black dashed horizontal lines.

No isolates harbored the *mcr* gene. The differences in expression of polymyxin B resistant genes between heteroresistant strains and reference strain (polymyxin B-susceptible *K. pneumoniae* ATCC 700603) were observed (Figure 2). The median fold changes in the expression of *phoP* and *phoQ* genes were 4.18 and 11.47, respectively (Figure 2). The increasing fold changes were also observed in *pmrA* and *pmrB* genes (5.78-fold and 2.31, respectively, Figure 2). As the negative regulator of PhoPQ, the fold change of *mgrB* decreased (0.12-fold) in all polymyxin B-heteroresistant strains. There is no significant overexpression of *pmrC* (0.79-fold) and *acrB* (0.90-fold) among polymyxin B-heteroresistant strains (Figure 2).

### The Resistant Subpopulation Can Survive Under Polymyxin B Pressure and Exist Stably Without Antibiotic

**FIGURE 3:**
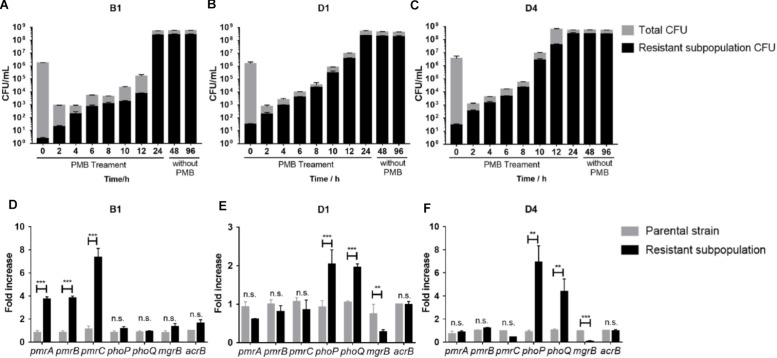
**(A–C)** Polymyxin B -resistant subpopulation CFU and total CFU of B1, D1, and D4 during 24 h treatment with 16 mg/L polymyxin B in liquid culture and subculture without polymyxin B for further 24 and 72 h (*n* = 3). **(D–F)** qRT-PCR analysis of the *phoP*, *phoQ*, *mgrB*, *pmrA*, *pmrB*, *pmrC*, and *acrB* gene expression in resistant and susceptible subpopulations of B1, D1, and D4. Relative abundance was calculated by normalizing the expression of each gene to the average expression of the housekeeping gene, *rpoB* (*n* = 4). Statistically significant: ^∗^*P* < 0.05, ^∗∗^*P* < 0.01, and ^∗∗∗^*P* < 0.001. n.s., not significantly different (unpaired *t*-test).

The stability of resistant subpopulations from three strains (B1, D1, and D4) was tested. As shown in Figures 3A–C, the resistant subpopulation could withstand and expand robustly under polymyxin B pressure, while most susceptible cells were killed over the first 2 h. After withdrawing polymyxin B, the resistant subpopulation still maintained a high level of polymyxin B resistance for 24 and 72 h, which suggested the resistant subpopulations can exist stably. Therefore, we speculated that this phenotypic change might be constant and correlated with genetic changes.

### Overexpressed PhoPQ or PmrAB System and Genetic Alternations in the Resistant Subpopulation

To determine the genetic alterations behind the stable heteroresistance phenotype, whole-genome sequencing (WGS) and quantitative PCR (qPCR) were performed on the paired parental strain and its resistant population. Differences in the transcript levels of *pmrCAB* operon between parental strain and a resistant population of B1 were observed (Figure 3D). The expression of *phoP* and *phoQ* increased in resistant populations compared to their expression in parental cells (Figures 3E,F). As the negative regulator of PhoPQ signaling, the expression of *mgrB* was lower in resistant populations (Figures 3E,F). There was no difference in the expression of *acrB* between parental strains and resistant subpopulations. We identified a mutation in *pmrA* that led to a missense variant (G53R) in polymyxin B-resistant cells of B1. There were some mutations in other genes (Supplementary Table S2), but it is unclear whether they might contribute to polymyxin B heteroresistance.

### Ceftazidime/avibactam Combinations Achieved Sustained Killing and Resistance Suppression

**FIGURE 4:**
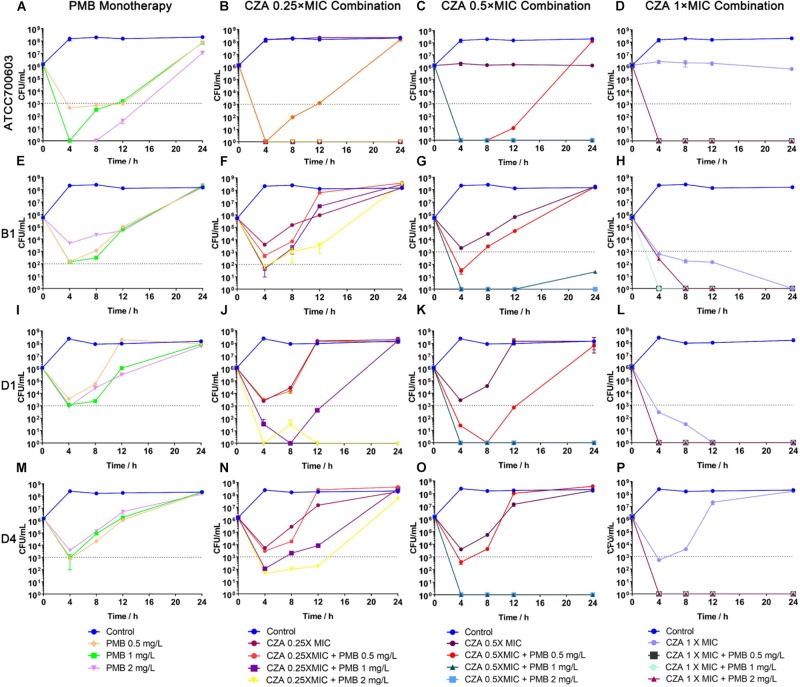
Time-kill curves for polymyxin B (PMB) and ceftazidime/avibactam (CZA) monotherapy and combination therapy against polymyxin B-susceptible *K. pneumoniae* ATCC 700603, and polymyxin B-heteroresistant *K. pneumoniae* (B1, D1, and D4). **(A,E,I,M)** PMB (0.5, 1, and 2 mg/L) as monotherapy; **(B,F,J,N)** PMB (0.5, 1 and 2 mg/L) in combination with CZA (0.25 × MIC); **(C,G,K,O)** PMB (0.5, 1 and 2 mg/L) in combination with CZA (0.5 × MIC); **(D,H,L,P)** PMB (0.5, 1 and 2 mg/L) in combination with CZA (1 × MIC). The limit of quantification is indicated by black dashed horizontal lines.

**FIGURE 5:**
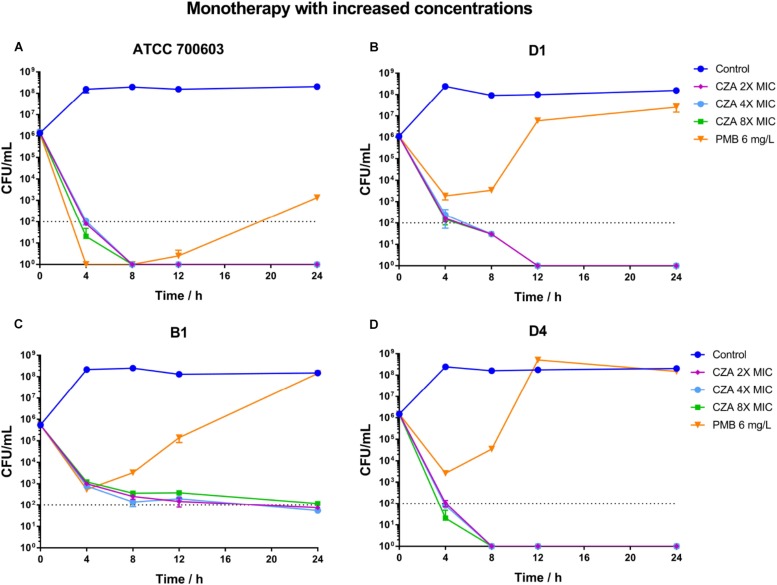
Time-kill curves for polymyxin B (PMB at 6 mg/L) and ceftazidime/avibactam (CZA at 2×, 4×, and 8× MIC) monotherapy with increased concentrations and against polymyxin B-susceptible *K. pneumoniae* ATCC 700603 **(A)**, and polymyxin B-heteroresistant *K. pneumoniae* B1 **(B)**, D1 **(C)**, and D4 **(D)**. The limit of quantification is indicated by black dashed horizontal lines.

Polymyxin B displayed a stronger and more sustained initial killing (≥3 log10 by 12 h) (Figure 4A and Supplementary Table S3) against susceptible isolate ATCC 700603, while all polymyxin B treatments only led to a ≥2 log10 reduction against heteroresistant isolates by 4 h and followed with bacterial re-growth (Figures 4E,I,M and Supplementary Table S3). This similar regrowth was observed even using supratherapeutic concentration against heteroresistant strains (6 mg/L) (Figure 5 and Supplementary Table S3). Ceftazidime/avibactam monotherapy showed concentration dependence. The higher concentrations (2×, 4×, and 8× MIC) displayed sustained bactericidal activity against all isolates over 24 h (Figure 5). In contrast, the bactericidal activity of ceftazidime/avibactam at lower concentrations (0.25×, 0.5×, and 1× MIC) varied and displayed a weaker effect.

The addition of ceftazidime/avibactam improved the efficacy of polymyxin B. Combining ceftazidime/avibactam at 0.25 × MIC with polymyxin B at 2 mg/L increased initial killing compared to that of monotherapy, but following regrowth was observed in B1 and D4 (Figures 4B,F,J,N). The addition of ceftazidime/avibactam at 0.25 × MIC with polymyxin B (1 mg/L) did not hamper the regrowth of heteroresistant isolates (Figures 4F,J,N). The killing effect of combination sustained for 24 h when combining ceftazidime/avibactam at 0.5 × MIC with polymyxin B (1 and 2 mg/L) (Figures  4C,G,K,O and Table 1). Colonies were undetectable over 24 h in all isolates when polymyxin B at all concentrations in combination with ceftazidime/avibactam at 1 × MIC, showing a rapid and durable bactericidal activity (Figures 4D,H,L,P), and synergy was observed (Table 1). To confirm the effect of combination, microbroth checkerboard assay was performed. The FICI values against heteroresistant strains can be seen in Table 1. Similarly, the synergistic effect of combination (FICI ≤ 0.5) was observed in B1 and D4, and the additive effect (FICI > 0.5 to ≤1) was observed in D1. Obviously, ceftazidime/avibactam strengthened the effect of polymyxin B and prevented the regrowth of polymyxin B-resistant cells. Additionally, combinations decreased the dose of each drug.

**TABLE 1:** The change in log_10_ CFU/ml at 4, 8, 12, and 48 h during time-kill experiments in combination with PMB and ceftazidime/avibactam^*a*^.

**Strains**	**MICs (mg/L)**		**FICI (*n* = 3)**	**Time (h)**	**Change in log_10_ CFU/ml**									
				
						**Ceftazidime/avibactam**			**Ceftazidime/avibactam**			**Ceftazidime/avibactam**		
	**PMB**	**CZA**			**Control**	**at 0.25 × MIC plus**			**at 0.5 × MIC plus**			**at 1 × MIC plus**		
						**PMB at (mg/L)**			**PMB at (mg/L)**			**PMB at (mg/L)**		
			
						**0.5**	**1**	**2**	**0.5**	**1**	**2**	**0.5**	**1**	**2**
ATCC700603	0.5	0.5	0.46 ± 0.12	4	2.06	**−6.13**	**−6.13**	**−6.13**	**−6.13**	**−6.13**	**−6.13**	**−6.13**	**−6.13**	**−6.13**
				8	2.16	**−4.16**	**−6.13**	**−6.13**	**−6.13**	**−6.13**	**−6.13**	**−6.13**	**−6.13**	**−6.13**
				12	2.06	**−3.03**	**−6.13**	**−6.13**	**−5.13**	**−6.13**	**−6.13**	**−6.13**	**−6.13**	**−6.13**
				24	2.18	2.05	**−6.13**	**−6.13**	2.00	**−6.13**	**−6.13**	**−6.13**	**−6.13**	**−6.13**
B1	HR	8	0.38 ± 0.10	4	2.59	**−3.03**	**−4.09**	**−3.93**	**−4.26**	**−5.74**	**−5.74**	**−3.34**	**−5.74**	**−5.74**
				8	2.66	–1.88	–2.43	–2.69	–2.30	**−5.74**	**−5.74**	**−5.74**	**−5.74**	**−5.74**
				12	2.37	2.06	0.96	–2.23	–1.06	**−5.74**	**−5.74**	**−5.74**	**−5.74**	**−5.74**
				24	2.44	2.84	2.73	2.75	2.50	**−5.74**	**−5.74**	**−5.74**	**−5.74**	**−5.74**
D1	HR	4	0.79 ± 0.16	4	2.34	–2.55	**−4.49**	**−6.04**	**−4.85**	**−6.04**	**−6.04**	**−6.04**	**−6.04**	**−6.04**
				8	1.92	**−1.84**	**−6.04**	**−4.56**	**−6.04**	**−6.04**	**−6.04**	**−6.04**	**−6.04**	**−6.04**
				12	1.95	2.15	**−3.39**	**−6.04**	**−3.20**	**−6.04**	**−6.04**	**−6.04**	**−6.04**	**−6.04**
				24	2.15	2.07	2.18	**−6.04**	1.80	**−6.04**	**−6.04**	**−6.04**	**−6.04**	**−6.04**
D4	HR	2	0.35 ± 0.13	4	2.21	–2.71	**−4.13**	**−4.44**	**−3.59**	**−6.18**	**−6.18**	**−6.18**	**−6.18**	**−4.88**
				8	2.03	–1.93	–2.88	**−4.18**	–2.55	**−6.18**	**−6.18**	**−6.18**	**−6.18**	**−6.18**
				12	2.07	2.23	–2.27	**−3.93**	1.84	**−6.18**	**−6.18**	**−6.18**	**−6.18**	**−6.18**
				24	2.14	2.46	2.33	1.52	2.39	**−6.18**	**−6.18**	**−6.18**	**−6.18**	**−6.18**

*^*a*^PMB, polymyxin B; CZA, ceftazidime/avibactam, ceftazidime/avibactam; HR, heteroresistance. Bactericidal activity (≥3 log10 CFU/ml reduction compared to the initial inoculum) is shown in bold. Additivity is defined as a reduction of between 1 and 2 log_10_ CFU/ml and synergy is defined as a reduction of ≥2 log_10_ CFU/ml caused by the combination compared to the results seen with the most active single agent in the combination. Additivity is highlighted with light gray shading and synergy with dark gray shading.*

## Discussion

Recently, many researchers pointed out that the clinically undetected heteroresistance might have a profound impact on treatment efficacy (Band et al., 2016, 2018; Band and Weiss, 2019). Polymyxin B has been widely used as a conventional lifesaver against superbugs for a long time, but the emergence of polymyxin heteroresistant will threaten the clinical use of polymyxins (Meletis et al., 2011; El-Halfawy and Valvano, 2015; Bardet et al., 2017; Wozniak et al., 2019). Therefore, it is critical to explore novel combination therapies which can delay or prevent the regrowth of polymyxin resistant subpopulations.

Here we evaluated the *in vitro* effect of the combination of polymyxin B with ceftazidime/avibactam against three polymyxin B-heteroresistant KPC-Kp (B1, D1, and D4). For polymyxin B-heteroresistant isolates, polymyxin B monotherapy resulted in a prompt killing effect, but followed by regrowth associated with the amplification of polymyxin B resistant subpopulations. The same situation had been reported in polymyxin B or colistin monotherapy against other Gram-negative bacteria (Ly et al., 2015; Lenhard et al., 2017; Zhao et al., 2017; Nicoloff et al., 2019). Therefore, many researchers concerned that resistant subpopulations might affect treament outcome (Band and Weiss, 2019). Ceftazidime/avibactam monotherapy at high concentrations (above 2 × MIC) prevented the regrowth of resistant subpopulations successfully. While ceftazidime/avibactam at low concentrations (<2 × MIC) showed relatively poorer effect and only delayed the regrowth of resistant subpopulations. Previous studies have tested polymyxins (polymyxin B or colistin) in combination with ceftazidime/avibactam against KPC-Kp with positive results (Nath et al., 2018; Mikhail et al., 2019). The similar killing effect against polymyxin B heteroresistant KPC-Kp was observed in our study. Nath et al. suggested that the addition of another antibiotic could be considered when ceftazidime/avibactam MIC values of isolates were close to the MIC breakpoint (Nath et al., 2018). Moreover, the addition of ceftazidime/avibactam improved the efficacy of polymyxin B and allowed for containment of all resistant subpopulations. Some animal models have demonstrated that heteroresistance might contribute to monotherapy treatment failure (Band et al., 2016, 2018). Our findings provided a potential polymyxin-based combination therapy, which held the promise to hamper the emergence of resistant subpopulations, and improved clinical outcomes in difficult to treat infections. The combination can also reduce the dose of both drugs. However, there might be some concerns about this combination therapy. Firstly, challenges in detection of polymyxin heteroresistance might set a barrier for clinicians to determine an appropriate time to start combination therapy. It is uncertain whether the combination therapy will still remain effective against heteroresistant strains after the failure of polymyxin B monotherapy. Secondly, a suitable dose of polymyxin B in combination need to be reevaluated due to its unreliably plasma concentrations in monotherapy (Bergen et al., 2015). Lastly, it is important to keep a balance between the theoretical benefits of combination therapy and worries that antibiotic combination will increase the financial burden and potentially more toxic than monotherapy.

Our study also showed that the microdilution broth method might misclassify heteroresistant strains as susceptible, which was consistent with other reports (Band et al., 2018; Turlej-Rogacka et al., 2018; Ezadi et al., 2019). There is a possibility that the resistant subpopulation is at a low frequency so that the growth cannot be detected by conventional tests. Different from persistency, which confer antibiotic tolerance at the cost of growth (Brauner et al., 2016), the resistant subpopulation can rapidly replicate in the presence of antibiotic (El-Halfawy and Valvano, 2015; Band et al., 2016, 2018; Anderson et al., 2018). Therefore, expanding the time of incubation may be helpful to detect the heteroresistance. Interestingly, some reports showed this increased resistance phenotype could revert from being entirely resistance to susceptible after removing antibiotic pressure (Band et al., 2016; Anderson et al., 2018). To our surprise, the resistant subpopulation in our study still dominated without selective pressure, even when the subculture time expanded. Later analysis demonstrated that several genetic changes might be responsible for it, involving the upregulation of PmrAB system and PhoPQ system and mutations. The changes in PmrAB system and PhoPQ system have been reported by other researches in different species (Jayol et al., 2015; Halaby et al., 2016; Charretier et al., 2018). Furthermore, *pmrA* G53R was detected in resistant cells of B1. The same mutation in *pmrA* have been described in colistin resistant *Enterobacter aerogenesc*, *S. enterica* and *K. pneumoniae* (Sun et al., 2009; Diene et al., 2013; Olaitan et al., 2014). Charretier et al. (2018) revealed that the mutations in the PmrAB regulatory pathway in *Acinetobacter baumannii*, which resulted in the overexpression of PmrAB system, led to colistin heteroresistance. Alterations in the PhoPQ turned out to be related with colistin heteroresistance in *K. pneumoniae* (Jayol et al., 2015). Except for the PmrAB and PhoPQ systems, mutations in the *lpxM* and *yciM* genes also played roles in the emergence of colistin-resistant *K. pneumoniae* (Halaby et al., 2016). In our study, no mutations in above genes were detected, but there were several genetic variations in other genes were detected in the resistant subpopulations of both D1 and D4. The roles of these mutations remained unclear and also need further experiments to confirm.

Nevertheless, this study had limitations. The sample size is small and may not be suitable for other KPC-producing strains. Antibiotic concentrations are constant and may not accurately reflect the real pharmacokinetics of antibiotics in a clinical dose. Therefore, pharmacodynamic activity need to be evaluated. The investigation of molecular mechanisms related to heteroresistance in *K. pneumoniae* were preliminary and need further exploration.

## Conclusion

In conclusion, our study provides evidence that the combination of ceftazidime/avibactam improved the antibacterial efficacy of polymyxin B against heteroresistant KPC-Kp and hindered the emergence of polymyxin resistant subpopulations. On top of that, an operational definition and uniform criteria for assessment of heteroresistant bacteria should be established to counteract heteroresistance.

## Ethics Statement

This study was approved by the Institutional Review Board of Second Affiliated Hospital of Soochow University. This study was retrospective and patients were not identified during data collection. Informed consent was not needed for this study.

## Author Contributions

XM participated in the design of the study, performed the antibiotic susceptibility tests, PAP analyses, qRT-PCR and time-kill assays, interpreted the data, and drafted the manuscript. YH carried out the qRT-PCR assays and participated in data analysis. XY and YC participated in whole-genome sequencing. JZ and LC participated in *de novo* assembly and SNPs analysis. RC collected the clinical strains. YL, CC, and BH designed the study, participated in data analysis, and provided critical revisions of the manuscript for important intellectual content.

## Conflict of Interest Statement

The authors declare that the research was conducted in the absence of any commercial or financial relationships that could be construed as a potential conflict of interest.
