# Supplementary material for: Ceftazidime/avibactam Improves the Antibacterial Efficacy of Polymyxin B Against Polymyxin B Heteroresistant KPC-2-Producing Klebsiella pneumoniae and Hinders Emergence of Resistant Subpopulation in vitro
Source: Front Microbiol. 2019 Sep 3;10:2029. doi: 10.3389/fmicb.2019.02029 (PMC6735287; doi:10.3389/fmicb.2019.02029)
Supplement: Supplementary file 1 [file Table_1.docx]

Table S1 Characteristics of seventeen KPC-Kp isolates

| isolates | β-Lactamase^a^ | MIC (mg/L) | | | | Outer member porin genotype | |
| --- | --- | --- | --- | --- | --- | --- | --- |
|  |  | CAZ | ceftazidime/avibactam | MEM | PB | *ompk35*^b^ | *ompk36*^c^ |
| A1 | KPC-2, OXA-10, SHV-11, TEM-30, CTX-M | 64 | 2/4 | 128 | 0.125 | Ala62fsX | Ins aa134-135 GD |
| A3 | KPC-2, OXA-1, SHV-11, TEM-30, CTX-M | 64 | 2/4 | 128 | － | Ala62fsX | Ins aa134-135 GD |
| A4 | KPC-2, SHV-11, TEM-30, CTX-M | 1024 | 4/4 | 128 | 0.125 | Ala62fsX | Ins aa134-135 GD |
| A7 | KPC-2, SHV-11, TEM-30, CTX-M | 64 | 2/4 | 128 | 0.125 | Ala62fsX | Ins aa134-135 GD |
| A9 | KPC-2, OXA-10, SHV-11, TEM-30, CTX-M | 64 | 2/4 | 128 | － | Ala62fsX | Ins aa134-135 GD |
| B1 | KPC-2, SHV, TEM-30, CTX-M | 512 | 8/4 | 256 | － | Ala62fsX | Ins aa134-135 GD |
| B2 | KPC-2, SHV, TEM-30, CTX-M | 512 | 4/4 | 128 | － | Ala62fsX | Ins aa134-135 GD |
| B8 | KPC-2, SHV, TEM-30, CTX-M | 512 | 2/4 | 256 | － | Ala62fsX | Ins aa134-135 GD |
| C6 | KPC-2, SHV-11, TEM-30, CTX-M | 64 | 2/4 | 128 | － | Ala62fsX | Ins aa134-135 GD |
| C8 | KPC-2, SHV-11, TEM-30, CTX-M | 64 | 2/4 | 128 | 0.125 | Ala62fsX | Ins aa134-135 GD |
| C9 | KPC-2, SHV-11, TEM-30, CTX-M | 1024 | 2/4 | 128 | 0.06125 | Ala62fsX | Ins aa134-135 GD |
| C10 | KPC-2, SHV-11, TEM-30, CTX-M | 64 | 4/4 | 128 | 0.125 | Ala62fsX | Ins aa134-135 GD |
| D1 | KPC-2, SHV, TEM-30, CTX-M | 512 | 4/4 | 256 | － | Ala62fsX | Ins aa134-135 GD |
| D3 | KPC-2, SHV, TEM-30, CTX-M | 512 | 2/4 | 128 | － | Ala62fsX | Ins aa134-135 GD |
| D4 | KPC-2, SHV, TEM-30, CTX-M | 1024 | 2/4 | 128 | － | Ala62fsX | Ins aa134-135 GD |
| D5 | KPC-2, SHV, TEM-30, CTX-M | 512 | 2/4 | 128 | 0.125 | Ala62fsX | Ins aa134-135 GD |
| D6 | KPC-2, SHV, TEM-30, CTX-M | 512 | 2/4 | 64 | － | Ala62fsX | Ins aa134-135 GD |

CAZ, ceftazidime; ceftazidime/avibactam, ceftazidime/avibactam; MEM, meropenem; PMB, polymyxin B; fsX, frameshift resulting in a premature stop codon; －, uninterpretable; ins, insertion; GD, GlyAsp.

^a^ Only carbapenemase and ESBL enzymes are listed.

^b^ The reference sequence of OmpK35 were based on an ST11 strain isolated from a Chinese hospital (GenBank accession number JX310555).

^c^ The reference sequence of OmpK36 were based on an ST11 strain isolated from a Chinese hospital (GenBank accession numbers JX291114).
