# Supplementary material for: Ceftazidime/avibactam Improves the Antibacterial Efficacy of Polymyxin B Against Polymyxin B Heteroresistant KPC-2-Producing Klebsiella pneumoniae and Hinders Emergence of Resistant Subpopulation in vitro
Source: Front Microbiol. 2019 Sep 3;10:2029. doi: 10.3389/fmicb.2019.02029 (PMC6735287; doi:10.3389/fmicb.2019.02029)
Supplement: Supplementary file 3 [file Table_3.docx]

Table S3 The change in log_10_ CFU/ml at 4, 8, 12 and 48 h during time-kill experiments with monotherapy with PMB or ceftazidime/avibactam.^a^

| Clinical isolate | Time(h) | Change in log_10_ CFU/ml | | | | | | | | | | | | |  |
| --- | --- | --- | --- | --- | --- | --- | --- | --- | --- | --- | --- | --- | --- | --- | --- |
|  |  | Control | Polymyxin B (mg/L) | | | |  | Ceftazidime/avibactam (mg/L) | | | | | | |  |
|  |  |  | 0.5 | 1 | 2 | 6 |  | 0.25×MIC | 0.5×MIC | 1×MIC | 2×MIC | 4×MIC | 8×MIC |  |  |
| ATCC 700603 | 4 | 2.06 | **-3.50** | **-6.13** | **-6.13** | **-6.13** |  | 2.05 | 0.16 | 0.29 | **-4.40** | **-6.13** | **-6.13** |  |  |
|  | 8 | 2.16 | **-3.30** | **-3.65** | **-6.13** | **-6.13** |  | 2.11 | 0.03 | 0.19 | **-3.97** | **-6.13** | **-6.13** |  |  |
|  | 12 | 2.06 | **-3.16** | -2.95 | **-4.54** | **-5.73** |  | 2.21 | 0.07 | 0.16 | **-3.30** | **-6.13** | **-6.13** |  |  |
|  | 24 | 2.18 | 1.75 | 1.73 | 0.91 | **-3.01** |  | 2.21 | 0.00 | -0.31 | **-6.13** | **-6.13** | **-6.13** |  |  |
| B1 | 4 | 2.59 | **-3.56** | **-3.59** | -2.09 | -3.01 |  | -2.14 | -2.43 | -2.96 | -2.84 | -2.74 | -2.65 |  |  |
|  | 8 | 2.66 | -2.68 | -3.26 | -1.39 | -2.22 |  | -0.55 | -1.30 | **-3.52** | **-3.59** | **-3.33** | **-3.18** |  |  |
|  | 12 | 2.37 | -0.74 | -0.92 | -1.06 | -0.59 |  | 0.24 | 0.06 | **-3.61** | **-3.44** | **-3.56** | **-3.16** |  | |
|  | 24 | 2.44 | 2.42 | 2.51 | 2.62 | 2.41 |  | 2.45 | 2.51 | **-5.74** | **-4.00** | **-3.87** | **-3.66** |  |  |
| D1 | 4 | 2.34 | -2.49 | -2.96 | **-3.11** | -2.77 |  | -2.65 | -2.61 | **-3.61** | **-3.82** | **-3.66** | **-3.88** |  |  |
|  | 8 | 1.92 | -1.30 | -2.67 | -1.62 | -2.51 |  | -1.59 | -1.47 | **-4.56** | **-4.56** | **-4.56** | **-4.56** |  |  |
|  | 12 | 1.95 | 2.27 | -0.01 | -0.55 | 0.73 |  | 2.16 | 2.15 | **-6.04** | **-6.04** | **-6.04** | **-6.04** |  |  |
|  | 24 | 2.15 | 1.96 | 1.91 | 1.75 | 1.37 |  | 2.26 | 2.14 | **-6.04** | **-6.04** | **-6.04** | **-6.04** |  |  |
| D4 | 4 | 2.21 | **-3.30** | **-3.21** | -2.61 | -2.76 |  | -2.46 | -2.58 | **-3.46** | **-4.15** | **-4.27** | **-4.70** |  |  |
|  | 8 | 2.03 | -1.85 | -1.23 | -1.04 | -1.63 |  | -0.74 | -1.44 | -2.59 | **-6.18** | **-6.18** | **-6.18** |  |  |
|  | 12 | 2.07 | -0.13 | 0.08 | 0.54 | 2.53 |  | 0.99 | 0.97 | 1.16 | **-6.18** | **-6.18** | **-6.18** |  | |
|  | 24 | 2.14 | 2.08 | 2.14 | 1.97 | 2.00 |  | 2.08 | 2.05 | 2.04 | **-6.18** | **-6.18** | **-6.18** |  |  |

^a^ Bactericidal activity (≥ 3 log10 CFU/ml reduction compared to the initial inoculum) is shown in bold.
